# *catena*-Poly[[bis­(nitrato-κ^2^ *O*,*O*′)barium]-bis­(μ-l-histidine-κ^3^ *O*,*O*′:*O*]

**DOI:** 10.1107/S1600536813027402

**Published:** 2013-10-12

**Authors:** P. Arularasan, G. Chakkaravarthi, R. Mohan

**Affiliations:** a Department of Physics, Presidency College, Chennai 600 005, India; b Department of Physics, CPCL Polytechnic College, Chennai 600 068, India

## Abstract

In the polymeric title compound, [Ba(NO_3_)_2_(C_6_H_9_N_3_O_2_)_2_]_*n*_, the Ba^II^ atom is located on a crystallographic twofold axis and is coordinated by ten O atoms. Six are derived from two zwitterionic l-histidine mol­ecules that simultaneously chelate one Ba^II^ atom and bridge to another. The remaining four O atoms are derived from two chelating nitrates. The mol­ecules assemble to form a chain along [010]. In the crystal, chains are linked *via* N—H⋯O and N—H⋯N hydrogen bonds, generating a three-dimensional network.

## Related literature

For the biological activity of histidine, see: Eichler *et al.* (2005 ▶); Wimalasena *et al.* (2007 ▶). For standard bond lengths, see: Allen *et al.* (1987 ▶). For related structures, see: Andra *et al.* (2010 ▶); Gokul Raj *et al.* (2006 ▶).
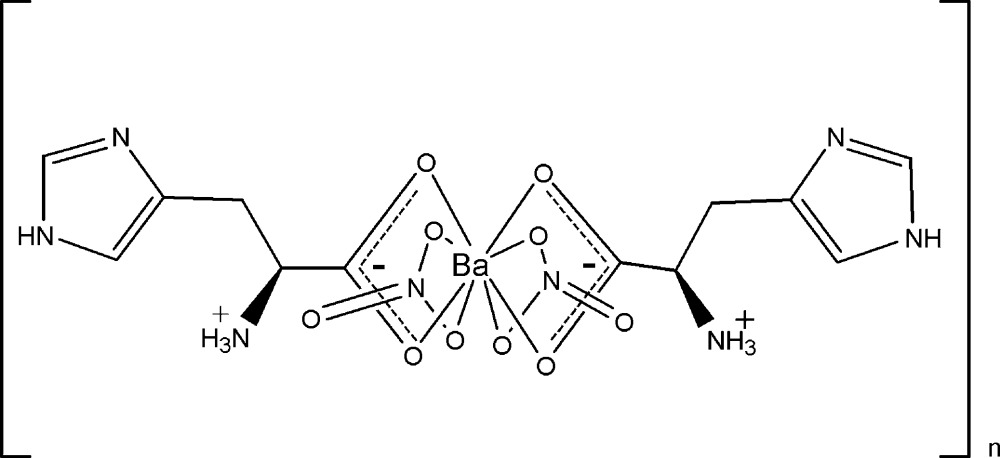


## Experimental

### 

#### Crystal data

[Ba(NO_3_)_2_(C_6_H_9_N_3_O_2_)_2_]
*M* *_r_* = 571.68
Monoclinic, 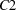

*a* = 24.9063 (8) Å
*b* = 4.7226 (1) Å
*c* = 8.3180 (3) Å
β = 105.432 (1)°
*V* = 943.11 (5) Å^3^
*Z* = 2
Mo *K*α radiation
μ = 2.18 mm^−1^
*T* = 295 K
0.18 × 0.14 × 0.12 mm

#### Data collection

Bruker Kappa APEXII diffractometer
Absorption correction: multi-scan (*SADABS*; Sheldrick, 1996 ▶) *T*_min_ = 0.695, *T*_max_ = 0.780
6598 measured reflections
3281 independent reflections
3281 reflections with *I* > 2σ(*I*)
*R*_int_ = 0.030

#### Refinement

*R*[*F* ^2^ > 2σ(*F* ^2^)] = 0.019
*wR*(*F* ^2^) = 0.048
*S* = 1.15
3281 reflections
142 parameters
2 restraints
H-atom parameters constrained
Δρ_max_ = 1.40 e Å^−3^
Δρ_min_ = −1.32 e Å^−3^
Absolute structure: Flack (1983 ▶), 1164 Friedel pairs
Absolute structure parameter: 0.004 (13)

### 

Data collection: *APEX2* (Bruker, 2004 ▶); cell refinement: *SAINT* (Bruker, 2004 ▶); data reduction: *SAINT*; program(s) used to solve structure: *SHELXS97* (Sheldrick, 2008 ▶); program(s) used to refine structure: *SHELXL97* (Sheldrick, 2008 ▶); molecular graphics: *PLATON* (Spek, 2009 ▶); software used to prepare material for publication: *SHELXL97*.

## Supplementary Material

Crystal structure: contains datablock(s) I, global. DOI: 10.1107/S1600536813027402/tk5259sup1.cif

Structure factors: contains datablock(s) I. DOI: 10.1107/S1600536813027402/tk5259Isup2.hkl

Supplementary material file. DOI: 10.1107/S1600536813027402/tk5259Isup3.cml

Additional supplementary materials:  crystallographic information; 3D view; checkCIF report

## Figures and Tables

**Table 1:** Hydrogen-bond geometry (Å, °)

*D*—H⋯*A*	*D*—H	H⋯*A*	*D*⋯*A*	*D*—H⋯*A*
N1—H1⋯O4^i^	0.86	2.29	2.854 (3)	123
N1—H1⋯O5^ii^	0.86	2.37	3.121 (3)	146
N3—H3*B*⋯O1^iii^	0.89	2.19	3.029 (2)	158
N3—H3*C*⋯O3^iv^	0.89	2.05	2.867 (3)	153
N3—H3*A*⋯N2^v^	0.89	1.94	2.827 (3)	174

Symmetry codes: (i) 
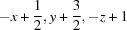
; (ii) 
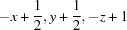
; (iii) 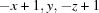; (iv) 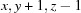; (v) 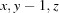.

**Fig. 1.**
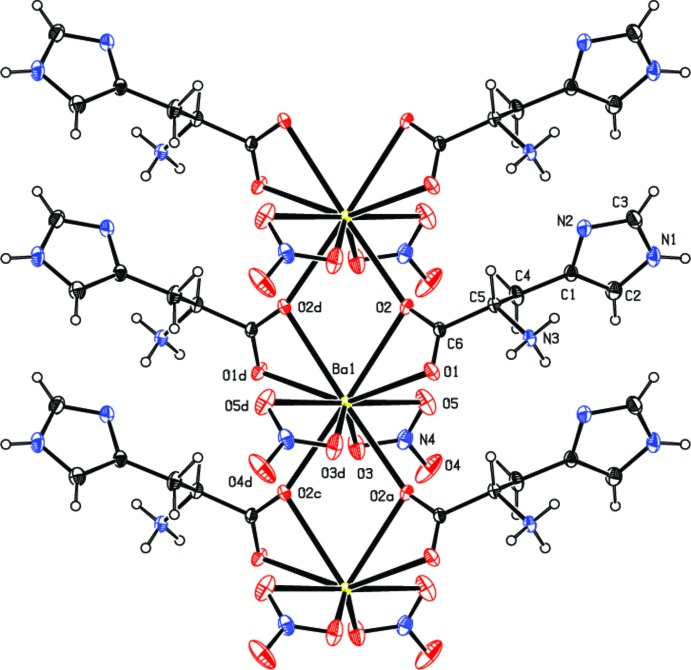
A portion of the polymeric structure in (I), with atom labels and 30% probability displacement ellipsoids for non-H atoms. The symmetry codes are : (a) x, -1+y, z; (c) 1-x, -1+y, 2-z; (d) 1-x, y, 2-z.

**Fig. 2.**
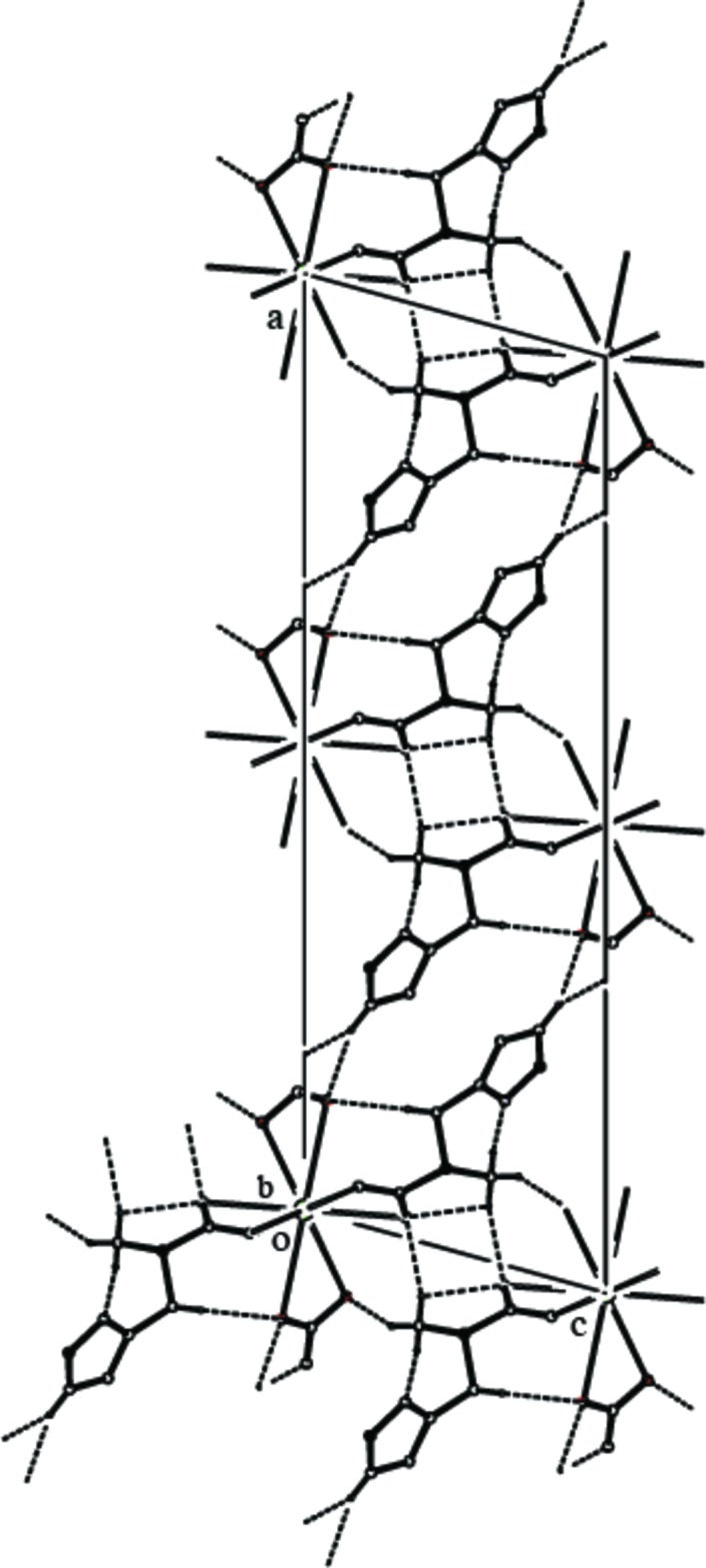
The packing of (I), viewed down the b axis. Intermolecular hydrogen bonds are shown as dashed lines. H atoms not involved in hydrogen bonding have been omitted.

## supplementary crystallographic information

### 1. Comment

Histidine derivatives are used in enzyme mechanisms and biomolecular interactions (Eichler *et al.*, 2005; Wimalasena *et al.*, 2007). We herein, report the crystal structure of the title compound (I), (Fig. 1). The geometric parameters of *L*-histidine moiety of (I) are comparable with the reported related structures (Andra *et al.*, 2010; Gokul Raj *et al.*, 2006).

The Ba atom sits on a crystallographic twofold axis and is co-ordinated by 10 O atoms, with six O atoms from carboxylate groups of *L*-Histidine and four O atoms from two nitrates. The Ba—O distances, ranging from 2.767 (5) to 2.966 (2) Å, are within normal range (Allen *et al.*, 1987).

The molecule coordinate through O to form a one dimensional chain, extending along [010]. The chains are interlinked through N—H···N and N—H···O hydrogen bonds (Table 1 & Fig. 2).

### 2. Experimental

The title compound was synthesized from the starting materials of *L* –histidine (3.1030 g) and barium nitrate (2.6133 g) which were taken in water. Single crystals suitable for X-ray diffraction were grown by the slow evaporation technique at room temperature.

### 3. Refinement

The C-bound H atoms were positioned geometrically and refined using a riding model with C—H = 0.93–0.98 Å, and with *U*_iso_(H) = 1.2*U*_eq_(C). For N-bound H atoms, N—H = 0.86–0.89 Å, and *U*_iso_(H) = 1.2–1.5 *U*_eq_(N). The components of anisotropic displacement parameters in the direction of the N1—C3 bond were restrained to be equal within an effective standard deviation of 0.001 using the DELU command in *SHELXL97* (Sheldrick, 2008). The maximum and minimum residual electron density peaks of 1.40 and -1.32 eÅ^-3^, respectively, were located 0.69 Å and 0.73 Å from the Ba atom, respectively.

### Crystal data

**Table:**

[Ba(NO_3_)_2_(C_6_H_9_N_3_O_2_)_2_]	*F*(000) = 564
*M**_r_* = 571.68	*D*_x_ = 2.013 Mg m^−^^3^
Monoclinic, *C*2	Mo *K*α radiation, λ = 0.71073 Å
Hall symbol: C 2y	Cell parameters from 6604 reflections
*a* = 24.9063 (8) Å	θ = 2.6–34.9°
*b* = 4.7226 (1) Å	µ = 2.18 mm^−^^1^
*c* = 8.3180 (3) Å	*T* = 295 K
β = 105.432 (1)°	Block, colourless
*V* = 943.11 (5) Å^3^	0.18 × 0.14 × 0.12 mm
*Z* = 2	

### Data collection

**Table:**

Bruker Kappa APEXII diffractometer	3281 independent reflections
Radiation source: fine-focus sealed tube	3281 reflections with *I* > 2σ(*I*)
Graphite monochromator	*R*_int_ = 0.030
ω and φ scan	θ_max_ = 34.9°, θ_min_ = 2.5°
Absorption correction: multi-scan (*SADABS*; Sheldrick, 1996)	*h* = −39→40
*T*_min_ = 0.695, *T*_max_ = 0.780	*k* = −6→7
6598 measured reflections	*l* = −13→11

### Refinement

**Table:**

Refinement on *F*^2^	Secondary atom site location: difference Fourier map
Least-squares matrix: full	Hydrogen site location: inferred from neighbouring sites
*R*[*F*^2^ > 2σ(*F*^2^)] = 0.019	H-atom parameters constrained
*wR*(*F*^2^) = 0.048	*w* = 1/[σ^2^(*F*_o_^2^) + (0.021*P*)^2^] where *P* = (*F*_o_^2^ + 2*F*_c_^2^)/3
*S* = 1.15	(Δ/σ)_max_ < 0.001
3281 reflections	Δρ_max_ = 1.40 e Å^−^^3^
142 parameters	Δρ_min_ = −1.32 e Å^−^^3^
2 restraints	Absolute structure: Flack (1983), 1164 Friedel pairs
Primary atom site location: structure-invariant direct methods	Absolute structure parameter: 0.004 (13)

### Special details

**Table:**

Geometry. All e.s.d.'s (except the e.s.d. in the dihedral angle between two l.s. planes) are estimated using the full covariance matrix. The cell e.s.d.'s are taken into account individually in the estimation of e.s.d.'s in distances, angles and torsion angles; correlations between e.s.d.'s in cell parameters are only used when they are defined by crystal symmetry. An approximate (isotropic) treatment of cell e.s.d.'s is used for estimating e.s.d.'s involving l.s. planes.
Refinement. Refinement of *F*^2^ against ALL reflections. The weighted *R*-factor *wR* and goodness of fit *S* are based on *F*^2^, conventional *R*-factors *R* are based on *F*, with *F* set to zero for negative *F*^2^. The threshold expression of *F*^2^ > σ(*F*^2^) is used only for calculating *R*-factors(gt) *etc*. and is not relevant to the choice of reflections for refinement. *R*-factors based on *F*^2^ are statistically about twice as large as those based on *F*, and *R*- factors based on ALL data will be even larger.

### Fractional atomic coordinates and isotropic or equivalent isotropic displacement parameters (Å^2^)

**Table:**

	*x*	*y*	*z*	*U*_iso_*/*U*_eq_	
C1	0.31466 (7)	0.2871 (5)	0.4170 (3)	0.0239 (4)	
C2	0.26202 (8)	0.1922 (6)	0.3475 (3)	0.0304 (4)	
H2	0.2451	0.0343	0.3795	0.036*	
C3	0.27774 (8)	0.5757 (17)	0.2181 (3)	0.0335 (5)	
H3	0.2722	0.7264	0.1436	0.040*	
C4	0.35756 (8)	0.1666 (6)	0.5623 (3)	0.0290 (5)	
H4A	0.3439	−0.0118	0.5935	0.035*	
H4B	0.3623	0.2948	0.6561	0.035*	
C5	0.41435 (6)	0.1163 (13)	0.5298 (2)	0.0215 (7)	
H5	0.4297	0.3001	0.5099	0.026*	
C6	0.45415 (7)	−0.0175 (5)	0.6835 (2)	0.0210 (3)	
N1	0.23922 (7)	0.3754 (6)	0.2217 (3)	0.0325 (4)	
H1	0.2062	0.3656	0.1559	0.039*	
N2	0.32423 (7)	0.5272 (4)	0.3354 (3)	0.0295 (5)	
N3	0.40970 (7)	−0.0632 (4)	0.3806 (2)	0.0228 (3)	
H3A	0.3830	−0.1914	0.3737	0.034*	
H3B	0.4420	−0.1508	0.3889	0.034*	
H3C	0.4014	0.0447	0.2896	0.034*	
N4	0.37614 (8)	−0.6072 (6)	1.0220 (3)	0.0332 (4)	
O1	0.47815 (7)	−0.2417 (4)	0.6626 (2)	0.0307 (3)	
O2	0.45918 (6)	0.1055 (13)	0.81973 (19)	0.0294 (4)	
O3	0.41918 (7)	−0.6466 (7)	1.1390 (3)	0.0470 (6)	
O4	0.33641 (9)	−0.7646 (8)	1.0069 (4)	0.0721 (10)	
O5	0.37673 (8)	−0.4073 (15)	0.9258 (3)	0.0556 (6)	
Ba1	0.5000	−0.4123	1.0000	0.01742 (4)	

### Atomic displacement parameters (Å^2^)

**Table:**

	*U*^11^	*U*^22^	*U*^33^	*U*^12^	*U*^13^	*U*^23^
C1	0.0190 (6)	0.0295 (10)	0.0223 (8)	0.0045 (7)	0.0037 (6)	−0.0027 (7)
C2	0.0230 (7)	0.0366 (11)	0.0318 (10)	0.0004 (7)	0.0078 (7)	−0.0016 (9)
C3	0.0314 (7)	0.0362 (14)	0.0296 (8)	0.0084 (12)	0.0024 (6)	0.004 (2)
C4	0.0245 (7)	0.0423 (15)	0.0203 (8)	0.0088 (7)	0.0060 (6)	0.0015 (7)
C5	0.0199 (5)	0.026 (2)	0.0174 (6)	0.0013 (9)	0.0026 (4)	0.0025 (9)
C6	0.0183 (6)	0.0243 (8)	0.0183 (7)	−0.0019 (6)	0.0015 (5)	0.0044 (6)
N1	0.0196 (6)	0.0449 (12)	0.0288 (9)	0.0058 (7)	−0.0007 (6)	−0.0050 (8)
N2	0.0245 (6)	0.0315 (14)	0.0288 (8)	0.0010 (6)	0.0006 (6)	−0.0009 (7)
N3	0.0214 (6)	0.0292 (9)	0.0173 (7)	0.0016 (6)	0.0043 (5)	0.0019 (6)
N4	0.0239 (7)	0.0451 (13)	0.0306 (10)	−0.0018 (8)	0.0074 (7)	−0.0103 (9)
O1	0.0340 (7)	0.0345 (9)	0.0228 (7)	0.0136 (7)	0.0059 (6)	0.0055 (6)
O2	0.0357 (5)	0.0258 (12)	0.0207 (5)	−0.0022 (12)	−0.0031 (4)	−0.0052 (12)
O3	0.0274 (7)	0.0762 (18)	0.0357 (10)	−0.0054 (9)	0.0056 (7)	0.0222 (11)
O4	0.0427 (11)	0.092 (2)	0.088 (2)	−0.0382 (13)	0.0288 (13)	−0.0513 (19)
O5	0.0467 (9)	0.0721 (16)	0.0462 (11)	0.020 (2)	0.0091 (8)	0.018 (3)
Ba1	0.01895 (5)	0.01937 (6)	0.01343 (5)	0.000	0.00342 (3)	0.000

### Geometric parameters (Å, º)

**Table:**

C1—C2	1.360 (3)	N3—H3B	0.8900
C1—N2	1.374 (3)	N3—H3C	0.8900
C1—C4	1.496 (3)	N4—O4	1.217 (3)
C2—N1	1.360 (4)	N4—O5	1.240 (6)
C2—H2	0.9300	N4—O3	1.255 (3)
C3—N2	1.320 (3)	N4—Ba1	3.271 (2)
C3—N1	1.353 (7)	O1—Ba1	2.8300 (17)
C3—H3	0.9300	O2—Ba1^i^	2.767 (5)
C4—C5	1.528 (3)	O2—Ba1	2.907 (5)
C4—H4A	0.9700	O3—Ba1	2.8022 (19)
C4—H4B	0.9700	O5—Ba1	2.9664 (18)
C5—N3	1.482 (4)	Ba1—O2^ii^	2.767 (5)
C5—C6	1.530 (3)	Ba1—O2^iii^	2.767 (5)
C5—H5	0.9800	Ba1—O3^iv^	2.8022 (19)
C6—O2	1.249 (4)	Ba1—O1^iv^	2.8300 (17)
C6—O1	1.251 (3)	Ba1—O2^iv^	2.907 (5)
C6—Ba1	3.180 (2)	Ba1—O5^iv^	2.9664 (18)
N1—H1	0.8600	Ba1—C6^iv^	3.180 (2)
N3—H3A	0.8900		
			
C2—C1—N2	109.63 (19)	O3—Ba1—O1^iv^	70.91 (6)
C2—C1—C4	128.2 (2)	O2^ii^—Ba1—O1	75.58 (9)
N2—C1—C4	122.15 (19)	O2^iii^—Ba1—O1	135.86 (7)
N1—C2—C1	106.0 (2)	O3^iv^—Ba1—O1	70.91 (6)
N1—C2—H2	127.0	O3—Ba1—O1	123.50 (5)
C1—C2—H2	127.0	O1^iv^—Ba1—O1	146.92 (9)
N2—C3—N1	110.5 (5)	O2^ii^—Ba1—O2^iv^	178.12 (13)
N2—C3—H3	124.8	O2^iii^—Ba1—O2^iv^	112.65 (5)
N1—C3—H3	124.8	O3^iv^—Ba1—O2^iv^	110.73 (6)
C1—C4—C5	114.35 (19)	O3—Ba1—O2^iv^	108.08 (8)
C1—C4—H4A	108.7	O1^iv^—Ba1—O2^iv^	45.61 (7)
C5—C4—H4A	108.7	O1—Ba1—O2^iv^	102.75 (7)
C1—C4—H4B	108.7	O2^ii^—Ba1—O2	112.65 (5)
C5—C4—H4B	108.7	O2^iii^—Ba1—O2	178.12 (14)
H4A—C4—H4B	107.6	O3^iv^—Ba1—O2	108.08 (8)
N3—C5—C4	111.5 (2)	O3—Ba1—O2	110.73 (6)
N3—C5—C6	110.6 (3)	O1^iv^—Ba1—O2	102.75 (7)
C4—C5—C6	109.95 (18)	O1—Ba1—O2	45.61 (7)
N3—C5—H5	108.2	O2^iv^—Ba1—O2	65.47 (13)
C4—C5—H5	108.2	O2^ii^—Ba1—O5^iv^	109.27 (11)
C6—C5—H5	108.2	O2^iii^—Ba1—O5^iv^	71.53 (14)
O2—C6—O1	125.7 (3)	O3^iv^—Ba1—O5^iv^	43.25 (9)
O2—C6—C5	116.9 (3)	O3—Ba1—O5^iv^	137.28 (10)
O1—C6—C5	117.4 (2)	O1^iv^—Ba1—O5^iv^	82.88 (6)
O2—C6—Ba1	66.1 (3)	O1—Ba1—O5^iv^	96.85 (7)
O1—C6—Ba1	62.55 (11)	O2^iv^—Ba1—O5^iv^	71.66 (13)
C5—C6—Ba1	160.70 (18)	O2—Ba1—O5^iv^	107.53 (13)
C3—N1—C2	107.9 (2)	O2^ii^—Ba1—O5	71.53 (14)
C3—N1—H1	126.0	O2^iii^—Ba1—O5	109.27 (11)
C2—N1—H1	126.0	O3^iv^—Ba1—O5	137.28 (10)
C3—N2—C1	105.9 (3)	O3—Ba1—O5	43.25 (9)
C5—N3—H3A	109.5	O1^iv^—Ba1—O5	96.85 (7)
C5—N3—H3B	109.5	O1—Ba1—O5	82.88 (6)
H3A—N3—H3B	109.5	O2^iv^—Ba1—O5	107.53 (13)
C5—N3—H3C	109.5	O2—Ba1—O5	71.66 (13)
H3A—N3—H3C	109.5	O5^iv^—Ba1—O5	179.1 (3)
H3B—N3—H3C	109.5	O2^ii^—Ba1—C6^iv^	158.50 (8)
O4—N4—O5	123.2 (3)	O2^iii^—Ba1—C6^iv^	91.99 (9)
O4—N4—O3	119.5 (3)	O3^iv^—Ba1—C6^iv^	115.73 (5)
O5—N4—O3	117.3 (2)	O3—Ba1—C6^iv^	91.67 (7)
O4—N4—Ba1	156.8 (2)	O1^iv^—Ba1—C6^iv^	23.09 (6)
O5—N4—Ba1	64.94 (12)	O1—Ba1—C6^iv^	125.88 (6)
O3—N4—Ba1	57.42 (12)	O2^iv^—Ba1—C6^iv^	23.14 (7)
C6—O1—Ba1	94.36 (13)	O2—Ba1—C6^iv^	86.16 (8)
C6—O2—Ba1^i^	143.9 (3)	O5^iv^—Ba1—C6^iv^	72.50 (9)
C6—O2—Ba1	90.8 (3)	O5—Ba1—C6^iv^	106.94 (10)
Ba1^i^—O2—Ba1	112.65 (5)	O2^ii^—Ba1—C6	91.99 (9)
N4—O3—Ba1	100.40 (16)	O2^iii^—Ba1—C6	158.50 (8)
N4—O5—Ba1	92.81 (15)	O3^iv^—Ba1—C6	91.67 (7)
O2^ii^—Ba1—O2^iii^	69.23 (15)	O3—Ba1—C6	115.73 (5)
O2^ii^—Ba1—O3^iv^	69.64 (6)	O1^iv^—Ba1—C6	125.88 (6)
O2^iii^—Ba1—O3^iv^	72.42 (8)	O1—Ba1—C6	23.09 (6)
O2^ii^—Ba1—O3	72.42 (8)	O2^iv^—Ba1—C6	86.16 (8)
O2^iii^—Ba1—O3	69.64 (6)	O2—Ba1—C6	23.14 (7)
O3^iv^—Ba1—O3	133.48 (13)	O5^iv^—Ba1—C6	106.94 (10)
O2^ii^—Ba1—O1^iv^	135.86 (7)	O5—Ba1—C6	72.50 (9)
O2^iii^—Ba1—O1^iv^	75.58 (9)	C6^iv^—Ba1—C6	108.20 (8)
O3^iv^—Ba1—O1^iv^	123.50 (5)		
			
N2—C1—C2—N1	0.3 (3)	C6—O2—Ba1—O1^iv^	178.66 (13)
C4—C1—C2—N1	178.7 (2)	Ba1^i^—O2—Ba1—O1^iv^	26.92 (7)
C2—C1—C4—C5	129.1 (3)	C6—O2—Ba1—O1	9.91 (11)
N2—C1—C4—C5	−52.6 (4)	Ba1^i^—O2—Ba1—O1	−141.84 (10)
C1—C4—C5—N3	−54.6 (4)	C6—O2—Ba1—O2^iv^	151.75 (17)
C1—C4—C5—C6	−177.8 (3)	Ba1^i^—O2—Ba1—O2^iv^	0.0
N3—C5—C6—O2	−176.0 (3)	C6—O2—Ba1—O5^iv^	92.20 (15)
C4—C5—C6—O2	−52.3 (4)	Ba1^i^—O2—Ba1—O5^iv^	−59.54 (9)
N3—C5—C6—O1	3.0 (3)	C6—O2—Ba1—O5	−88.25 (17)
C4—C5—C6—O1	126.7 (3)	Ba1^i^—O2—Ba1—O5	120.00 (11)
N3—C5—C6—Ba1	−81.9 (5)	C6—O2—Ba1—C6^iv^	162.49 (12)
C4—C5—C6—Ba1	41.8 (9)	Ba1^i^—O2—Ba1—C6^iv^	10.74 (6)
N2—C3—N1—C2	0.4 (4)	Ba1^i^—O2—Ba1—C6	−151.75 (17)
C1—C2—N1—C3	−0.4 (3)	N4—O5—Ba1—O2^ii^	69.8 (3)
N1—C3—N2—C1	−0.3 (4)	N4—O5—Ba1—O2^iii^	10.7 (3)
C2—C1—N2—C3	0.0 (3)	N4—O5—Ba1—O3^iv^	95.3 (2)
C4—C1—N2—C3	−178.6 (3)	N4—O5—Ba1—O3	−13.90 (19)
O2—C6—O1—Ba1	20.7 (3)	N4—O5—Ba1—O1^iv^	−66.4 (3)
C5—C6—O1—Ba1	−158.23 (19)	N4—O5—Ba1—O1	146.9 (3)
O1—C6—O2—Ba1^i^	112.2 (4)	N4—O5—Ba1—O2^iv^	−111.9 (3)
C5—C6—O2—Ba1^i^	−68.9 (4)	N4—O5—Ba1—O2	−167.6 (3)
Ba1—C6—O2—Ba1^i^	132.2 (3)	N4—O5—Ba1—C6^iv^	−87.7 (3)
O1—C6—O2—Ba1	−20.0 (3)	N4—O5—Ba1—C6	168.1 (3)
C5—C6—O2—Ba1	158.9 (2)	O2—C6—Ba1—O2^ii^	154.08 (15)
O4—N4—O3—Ba1	153.1 (2)	O1—C6—Ba1—O2^ii^	−44.19 (13)
O5—N4—O3—Ba1	−26.4 (3)	C5—C6—Ba1—O2^ii^	50.8 (6)
O4—N4—O5—Ba1	−155.0 (3)	O2—C6—Ba1—O2^iii^	−177.57 (18)
O3—N4—O5—Ba1	24.4 (3)	O1—C6—Ba1—O2^iii^	−15.8 (2)
N4—O3—Ba1—O2^ii^	−67.5 (2)	C5—C6—Ba1—O2^iii^	79.2 (6)
N4—O3—Ba1—O2^iii^	−141.3 (2)	O2—C6—Ba1—O3^iv^	−136.24 (14)
N4—O3—Ba1—O3^iv^	−104.1 (2)	O1—C6—Ba1—O3^iv^	25.49 (14)
N4—O3—Ba1—O1^iv^	137.5 (2)	C5—C6—Ba1—O3^iv^	120.5 (6)
N4—O3—Ba1—O1	−9.1 (2)	O2—C6—Ba1—O3	82.67 (16)
N4—O3—Ba1—O2^iv^	110.6 (2)	O1—C6—Ba1—O3	−115.60 (14)
N4—O3—Ba1—O2	40.7 (2)	C5—C6—Ba1—O3	−20.6 (6)
N4—O3—Ba1—O5^iv^	−167.1 (2)	O2—C6—Ba1—O1^iv^	−1.61 (15)
N4—O3—Ba1—O5	14.0 (2)	O1—C6—Ba1—O1^iv^	160.12 (10)
N4—O3—Ba1—C6^iv^	127.2 (2)	C5—C6—Ba1—O1^iv^	−104.9 (6)
N4—O3—Ba1—C6	16.0 (2)	O2—C6—Ba1—O1	−161.7 (2)
C6—O1—Ba1—O2^ii^	134.00 (14)	C5—C6—Ba1—O1	95.0 (6)
C6—O1—Ba1—O2^iii^	171.74 (13)	O2—C6—Ba1—O2^iv^	−25.57 (16)
C6—O1—Ba1—O3^iv^	−152.92 (15)	O1—C6—Ba1—O2^iv^	136.16 (13)
C6—O1—Ba1—O3	76.98 (16)	C5—C6—Ba1—O2^iv^	−128.8 (6)
C6—O1—Ba1—O1^iv^	−30.33 (12)	O1—C6—Ba1—O2	161.7 (2)
C6—O1—Ba1—O2^iv^	−45.12 (14)	C5—C6—Ba1—O2	−103.3 (6)
C6—O1—Ba1—O2	−9.93 (12)	O2—C6—Ba1—O5^iv^	−95.07 (16)
C6—O1—Ba1—O5^iv^	−117.80 (18)	O1—C6—Ba1—O5^iv^	66.65 (17)
C6—O1—Ba1—O5	61.31 (18)	C5—C6—Ba1—O5^iv^	161.7 (6)
C6—O1—Ba1—C6^iv^	−44.47 (16)	O2—C6—Ba1—O5	84.17 (19)
C6—O2—Ba1—O2^ii^	−28.25 (17)	O1—C6—Ba1—O5	−114.11 (18)
Ba1^i^—O2—Ba1—O2^ii^	180.000 (1)	C5—C6—Ba1—O5	−19.1 (6)
C6—O2—Ba1—O3^iv^	46.66 (15)	O2—C6—Ba1—C6^iv^	−18.42 (12)
Ba1^i^—O2—Ba1—O3^iv^	−105.09 (7)	O1—C6—Ba1—C6^iv^	143.31 (14)
C6—O2—Ba1—O3	−107.18 (14)	C5—C6—Ba1—C6^iv^	−121.7 (6)
Ba1^i^—O2—Ba1—O3	101.07 (8)		

Symmetry codes: (i) *x*, *y*+1, *z*; (ii) *x*, *y*−1, *z*; (iii) −*x*+1, *y*−1, −*z*+2; (iv) −*x*+1, *y*, −*z*+2.

### Hydrogen-bond geometry (Å, º)

**Table:**

*D*—H···*A*	*D*—H	H···*A*	*D*···*A*	*D*—H···*A*
N1—H1···O4^v^	0.86	2.29	2.854 (3)	123
N1—H1···O5^vi^	0.86	2.37	3.121 (3)	146
N3—H3*B*···O1^vii^	0.89	2.19	3.029 (2)	158
N3—H3*C*···O3^viii^	0.89	2.05	2.867 (3)	153
N3—H3*A*···N2^ii^	0.89	1.94	2.827 (3)	174

Symmetry codes: (ii) *x*, *y*−1, *z*; (v) −*x*+1/2, *y*+3/2, −*z*+1; (vi) −*x*+1/2, *y*+1/2, −*z*+1; (vii) −*x*+1, *y*, −*z*+1; (viii) *x*, *y*+1, *z*−1.
